# eLIFEwithIBD: study protocol for a randomized controlled trial of an online acceptance and commitment therapy and compassion-based intervention in inflammatory bowel disease

**DOI:** 10.3389/fpsyg.2024.1369577

**Published:** 2024-08-09

**Authors:** Cláudia Ferreira, Joana Pereira, Inês Matos-Pina, David Skvarc, Ana Galhardo, Nuno Ferreira, Sérgio A. Carvalho, Paola Lucena-Santos, Bárbara S. Rocha, Sara Oliveira, Francisco Portela, Inês A. Trindade

**Affiliations:** ^1^CINEICC, Faculty of Psychology and Education Sciences, University of Coimbra, Coimbra, Portugal; ^2^School of Psychology, Deakin University, Geelong, VIC, Australia; ^3^Instituto Superior Miguel Torga, Coimbra, Portugal; ^4^School of Social Sciences, University of Nicosia, Nicosia, Cyprus; ^5^HEI-Lab: Digital Human-Environment Interaction Lab, School of Psychology and Life Sciences, Lusófona University, Lisbon, Portugal; ^6^Center for Neuroscience and Cell Biology, Faculty of Pharmacy, University of Coimbra, Coimbra, Portugal; ^7^Gastroenterology Service, Coimbra University Hospital (CHUC), Coimbra, Portugal; ^8^EMBRACE Lab, Center for Health and Medical Psychology (CHAMP), School of Behavioural, Social and Legal Sciences, University of Örebro, Örebro, Sweden

**Keywords:** acceptance and commitment therapy, compassion, inflammatory bowel disease, mindfulness, randomized controlled trial, study protocol

## Abstract

**Background:**

Inflammatory bowel disease (IBD) entails physical, psychological, and social burden and holds a significant impact on quality of life. Experiential avoidance, cognitive fusion, shame, and self-criticism have been identified as possible therapeutic targets for improving mental health in people with IBD. Traditional face-to-face psychological therapy continues to provide obstacles for patients seeking assistance. Online psychological therapies centered on acceptance, mindfulness, and compassion have been shown to improve psychological distress in other populations.

**Objective:**

This paper presents the study protocol of a two-arm Randomized Controlled Trial (RCT) of an ACT and compassion-based, online intervention – eLIFEwithIBD - on the improvement of psychological distress, quality of life, work and social functioning, IBD symptom perception, illness-related shame, psychological flexibility, and self-compassion.

**Methods:**

The eLIFEwithIBD intervention is an adaptation of the LIFEwithIBD programme (delivered through an in-person group format) and entails an ACT, mindfulness, and compassion-based intervention designed to be delivered as an e-health tool for people with IBD. This protocol outlines the structure and contents of the eLIFEwithIBD intervention. Participants were recruited by an advertisement on the social media platforms of Portuguese Associations for IBD in January 2022. A psychologist conducted a brief interview with 80 patients who were interested in participating. Fifty-five participants were selected and randomly assigned to one of two conditions [experimental group (eLIFEwithIBD + medical TAU; *n* = 37) or control group (medical TAU; *n* = 18)]. Outcome measurement took place at baseline, post-intervention, and 4-month follow-up. All analyses are planned as intent-to-treat (ITT).

**Results:**

The eLIFEwithIBD intervention is expected to empower people with IBD by fostering psychological strategies that promote illness adjustment and well-being and prevent subsequent distress. The eLIFEwithIBD aims to gain a novel and better understanding of the role of online contextual behavioral interventions on improving the quality of life and mental health of people with IBD.

**Clinical Trial Registration:**

https://classic.clinicaltrials.gov/ct2/show/NCT05405855, NCT05405855.

## 1 Introduction

Inflammatory bowel disease (IBD) is a group of chronic, relapsing, and remittent inflammatory conditions of the gastrointestinal tract (Crohn’s disease and ulcerative colitis). According to epidemiological studies, at least 3.7 million people are living with IBD worldwide (with 30.000 new cases each year only in Europe) (Ng et al., 2017; Bernstein et al., 2019). IBD holds a substantial impact on quality of life (QoL) (Alatab et al., 2020). Due to the characteristics and severity of symptoms, the treatment, and the uncertainty of the prognosis, the daily life of people with IBD may be significantly affected by the disease burden (Rocchi et al., 2012; Devlen et al., 2014; Bernstein et al., 2019), with impaired functioning (Wolfe and Sirois, 2008), and high psychological distress (Graff et al., 2009; Mikocka-Walus et al., 2016).

People living with IBD tend to report significantly higher rates of anxiety and depression compared to the general population (Mikocka-Walus et al., 2016; Byrne et al., 2017), and the literature highlights that, in addition to medical treatment, IBD management should also consider psychosocial aspects (IBD Standards Group, 2013). Several different psychological interventions have been tested in this population, with promising results (Bennebroek Evertsz et al., 2017; Stapersma et al., 2018), with mindfulness and acceptance-based interventions, such as Acceptance and Commitment Therapy (ACT), being the focus of recent growing attention (Ungaro et al., 2017; Ferreira, 2019) and providing approaches theoretically consistent with findings on the drivers of psychological distress in IBD (Trindade et al., 2017a,b, 2018; Wynne et al., 2019).

ACT aims to promote psychological flexibility (Trindade et al., 2019), defined as the ability to alter or maintain one’s behavior in accordance with one’s values and objectives while being aware of and open to internal experiences (e.g., thoughts, emotions, physical sensations) (Kashdan and Rottenberg, 2010). ACT uses acceptance and mindfulness practices with commitment and behavior change strategies. ACT has shown efficacy in a wide range of health conditions (e.g., depression, anxiety, chronic pain), being generally superior to inactive controls, treatment as usual, and most active intervention conditions (Hayes et al., 2006; A-Tjak et al., 2015). There is growing evidence that ACT may help people with IBD cope with symptoms and psychological distress, particularly in the early stages of the illness (Ungaro et al., 2017; Gloster et al., 2020).

Given that people with IBD are prone to experience disease-related stigma (Dober et al., 2020), shame, and self-criticism (Keefer and Taft, 2016), it has been suggested that compassion-based approaches may be useful in this context as well (Trindade et al., 2019). These approaches acknowledge that all humans go through difficult, uncertain, and painful experiences, and advocate that those experiences should be welcomed with kindness and caring action (Neff, 2011; Gilbert, 2014; Trindade et al., 2020a,b). Compassion may be an attribute defined as “a sensitivity to suffering in self and others with a commitment to try to alleviate and prevent it” (Trindade et al., 2020a,b, p. 19), that may aid individuals in accepting and coping with the hardships of a long-term medical condition (Ashworth et al., 2015; Strauss et al., 2016; Gooding et al., 2020; Austin et al., 2021). The efficacy of compassion-based interventions to decrease psychological distress and psychopathology and improve quality of life has obtained increasing evidence (Hudson et al., 2020). Self-compassion has been shown to be a protective factor against stress, depressive symptoms, and anxiety (Kirby et al., 2017). However, to our knowledge, no compassion-based intervention has yet been tested for IBD.

According to previous research, psychosocial indicators have been shown to be improved through the integration of self-compassion, acceptance and mindfulness components in interventions applied to a variety of health conditions (e.g., cancer, chronic medical illness, pain) and seems to be feasible and effective (Brown et al., 2019; Gooding et al., 2020; Austin et al., 2021; Trindade and Sirois, 2021).

The availability of psychological interventions in IBD healthcare may be limited (Trindade et al., 2020a,b), and traditional face-to-face interventions still generally face relevant obstacles to implementation and adherence (such as stigma, time constraints, lack of transportation) (Marín-Jiménez et al., 2017). Developments in eHealth approaches have provided alternative forms of psychological treatment delivery by facilitating access to specialized mental healthcare services that do not present time and transportation constraints (Marks et al., 2007). Additionally, online therapy has several advantages: (a) reduced waiting lists; (b) 24-h availability; (c) overcomes geographic obstacles in treatment access; (d) ensures anonymity, thus overcoming stigma and shame; (e) access to specialized treatment; (f) cost-effectiveness (Cuijpers et al., 2008; Andersson and Titov, 2014; Donker et al., 2015; Ebert et al., 2015; Walker et al., 2015; Trindade et al., 2021a,b,c,Tsiouris et al., 2021). Online therapy is usually assessed as acceptable by patients (ten Have et al., 2013; Arjadi et al., 2018), and may be a preferred method over face-to-face formats (Gun et al., 2011). On the other hand, online psychotherapy can be associated with low adherence rates, particularly when it is self-guided (Wahbeh et al., 2014). ACT (Karyotaki et al., 2015; Lin et al., 2017; Trindade et al., 2021a,b,c), mindfulness (van de Graaf et al., 2021), and compassion-based (Liu et al., 2022) interventions delivered online seem suitable and efficacious in improving psychological outcomes in different chronic health conditions. As IBD tends to have an early onset (15–30 years) and lifetime treatment, IBD represents an appropriate setting for the development and dissemination of eHealth interventions, given this population is most proficient in the use of platforms and apps (Loftus, 2004; Carvalho et al., 2021). To our knowledge, no previous studies examined the efficacy online integrative ACT, mindfulness, and compassion interventions in IBD.

The present study aims to examine the acceptability, usability, and preliminary efficacy of an online intervention – the eLIFEwithIBD intervention - on the improvement of psychological distress and other psychosocial indicators in IBD. This intervention was be adapted from a face-to-face group intervention (LIFEwithIBD) (Yin and Neyens, 2020) previously tested in patients with IBD, and is the first to incorporate ACT, mindfulness and compassion components through an online intervention targeting this population. This intervention (eLIFEwithIBD + Treatment and Usual [TAU])‘s superiority in improving psychological distress, quality of life, work and social functioning, IBD symptom perception, illness-related shame, psychological flexibility, and self-compassion will be compared to TAU. This paper presents the structure and contents of the eLIFEwithIBD intervention as well as the design of this Randomized Controlled Trial (RCT).

## 2 Methods

### 2.1 Participants (inclusion and exclusion criteria)

Inclusion criteria for this study were: (a) Portuguese adults aged between 18 and 65 years old; (b) confirmed diagnosis of IBD (at least for 6 months); (c) regular access to computers and internet; (d) able to write and read Portuguese; (e) informed consent.

Exclusion criteria included: (a) psychiatric disorder diagnosis (major depressive disorder, psychotic disorder, bipolar disorder, substance abuse) or suicidal ideation (assessed by the Patient Health Questionnaire-9 [PHQ-9]) (Trindade et al., 2021a,b,c); (b) current psychological treatment; (c) and pregnancy.

Participants who were not eligible to participate in the study due to the first exclusion criteria were given feedback and advised to seek specialized treatment or psychological support.

### 2.2 Recruitment and data collection

This trial is registered at ClinicalTrials.gov (Identifier: NCT05405855). The planning and execution followed the American Psychological Association (Ferreira et al., 2019) and the World Medical Association’s Declaration of Helsinki (American Psychological Association, 1992) guidelines. Authorization for sample recruitment was obtained from the Faculty of Psychology and Education Sciences of the University of Coimbra. Recruitment was facilitated by advertisements on social media by Portuguese Associations for IBD in January 2022, which included a link to a registration questionnaire. Individuals interested in participating were screened by a psychologist through a short interview (to present the aims of the study, obtain informed consent, and assess eligibility), conducted by videoconference or phone call. Eligible participants were asked to complete baseline questionnaires (T0) via an online survey.

**Figure 1:**
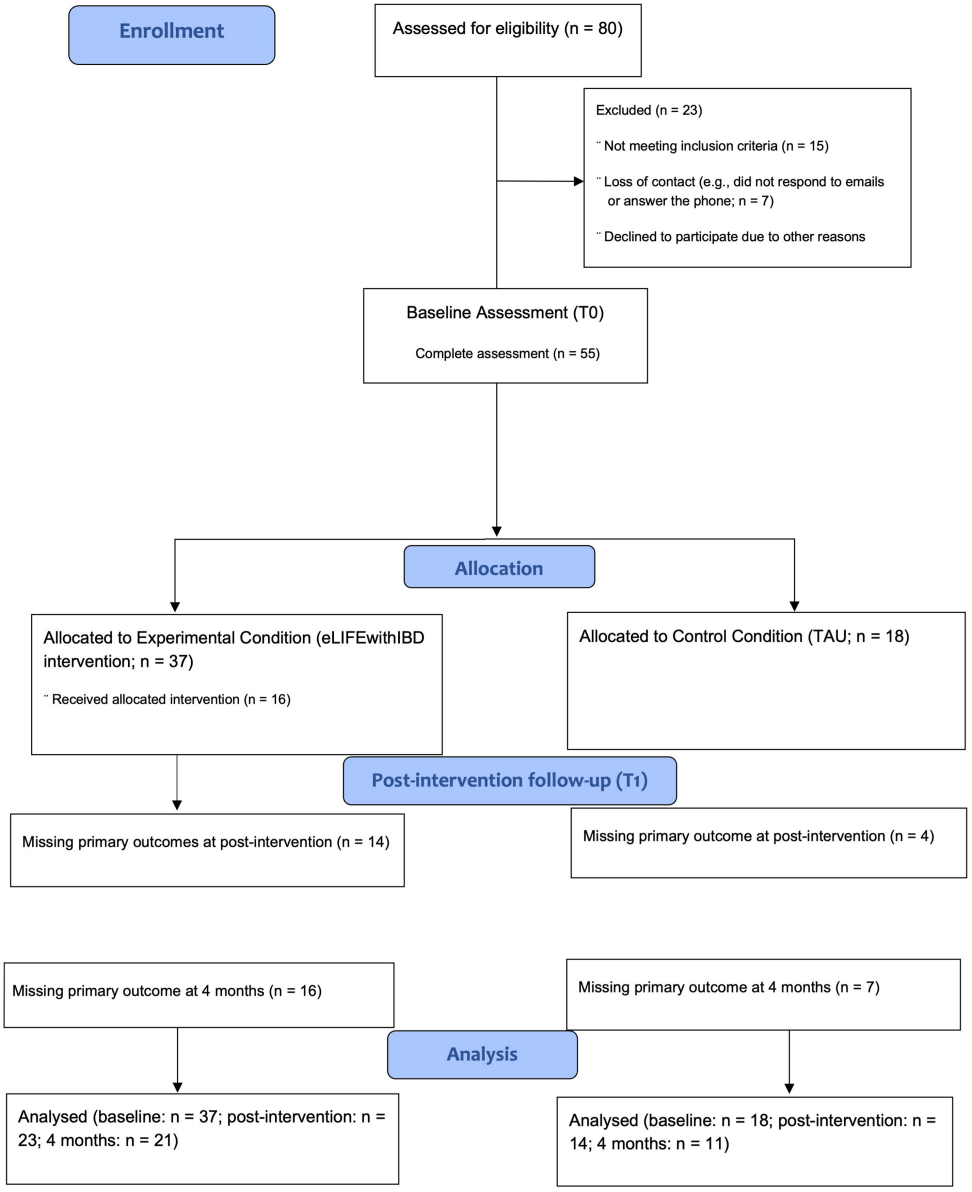
Preliminary CONSORT 2010 flow diagram.

After completing the baseline assessment (T0), participants were randomly assigned (computer-generated random allocation, ratio 2:1) (World Medical Association, 2013) to the experimental condition (eLIFEwithIBD intervention + TAU) or the control condition (TAU). Participants in both conditions were informed about their assigned treatment condition and then, participants in the experimental condition had access to the eLIFEwithIBD platform. After the intervention (T1) and in the 4-month (T2) follow-up, participants in both conditions received notifications via email to complete self-report questionnaires again, via an online survey. Participants in the control condition will receive the intervention after completing the last assessment of the study (T2). Figure 1 shows the CONSORT flow diagram.

### 2.3 The eLIFEwithIBD intervention

The eLIFEwithIBD intervention is an adaptation of the LIFEwithIBD intervention (delivered in an in-person group format) (Yin and Neyens, 2020), which in turn was based on a face-to-face intervention for people with cancer (Brown et al., 2019). The eLIFEwithIBD intervention is an ACT, mindfulness, and compassion-based intervention designed to be delivered as an e-health tool for people with IBD. The online eLIFEwithIBD intervention focuses on education regarding IBD, the functioning of the human mind, emotions, and fatigue, promotion of willingness and acceptance of internal experiences, values clarification, and promotion of committed action, mindfulness, compassion, and gratitude.

#### 2.3.1. Onboarding

**Figure 2:**
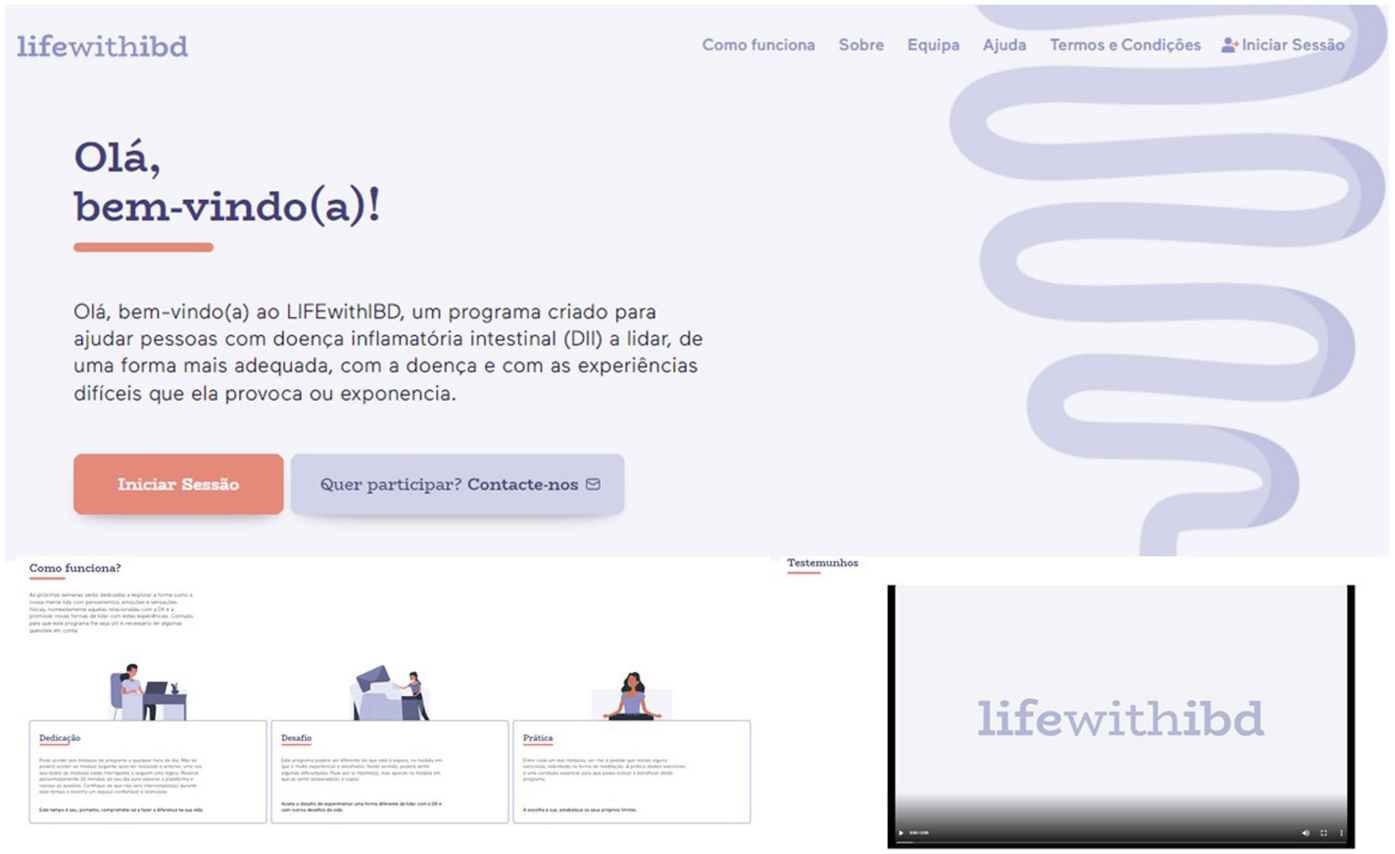
Screenshots of the homepage.

When participants access the eLIFEwithIBD platform, they are presented with a homepage containing the program title, a welcome message, a brief explanation of how the program works, and video testimonies of participants who completed the program in the face-to-face format. Participants also have access to tabs with diverse information about the platform and intervention (e.g., how the intervention works, team, help, terms and conditions) (Figure 2) and a button that directs them to the Intervention’s sessions.

Participants from the experimental group received an email with an invitation to register in the platform, in the beginning of the study. After registering, they could login in the platform via the “Sign in” button and start the sessions.

**Figure 3:**
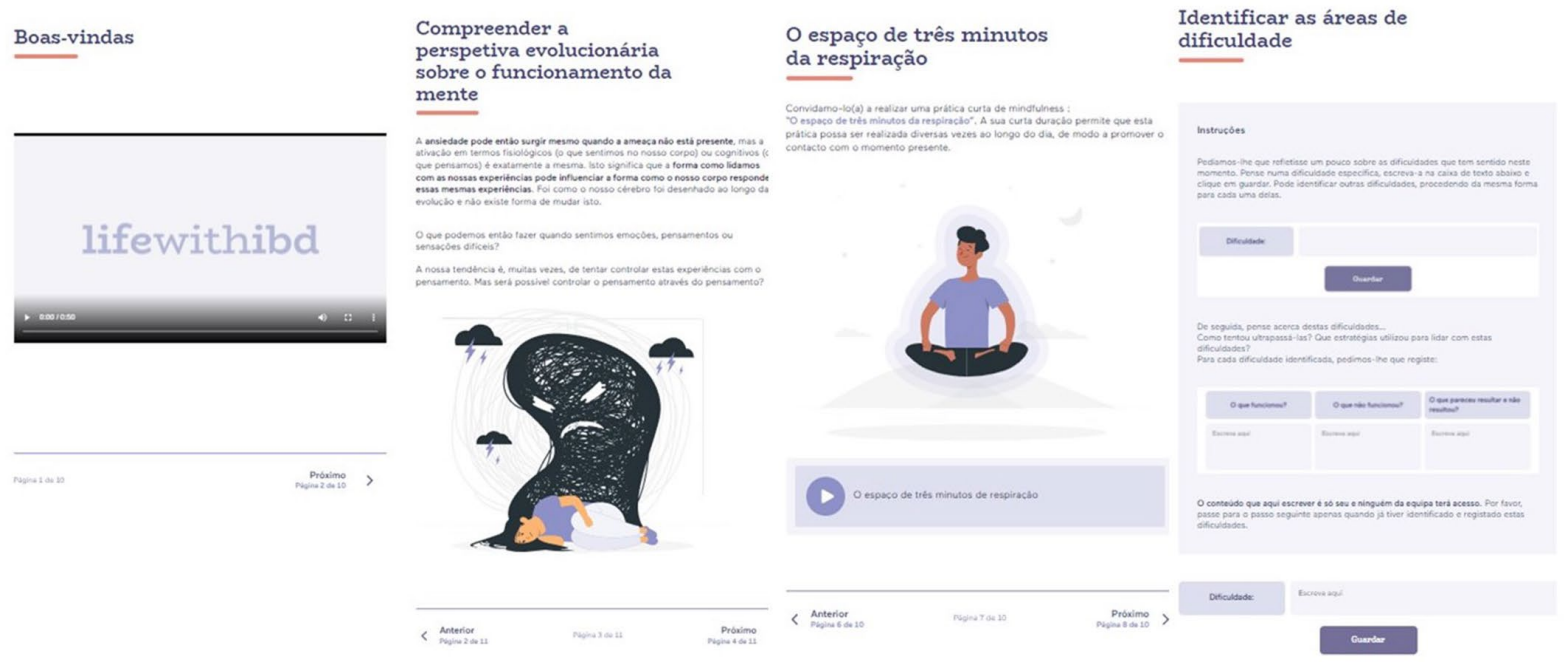
Presentation of the content of the sessions.

**Figure 4:**
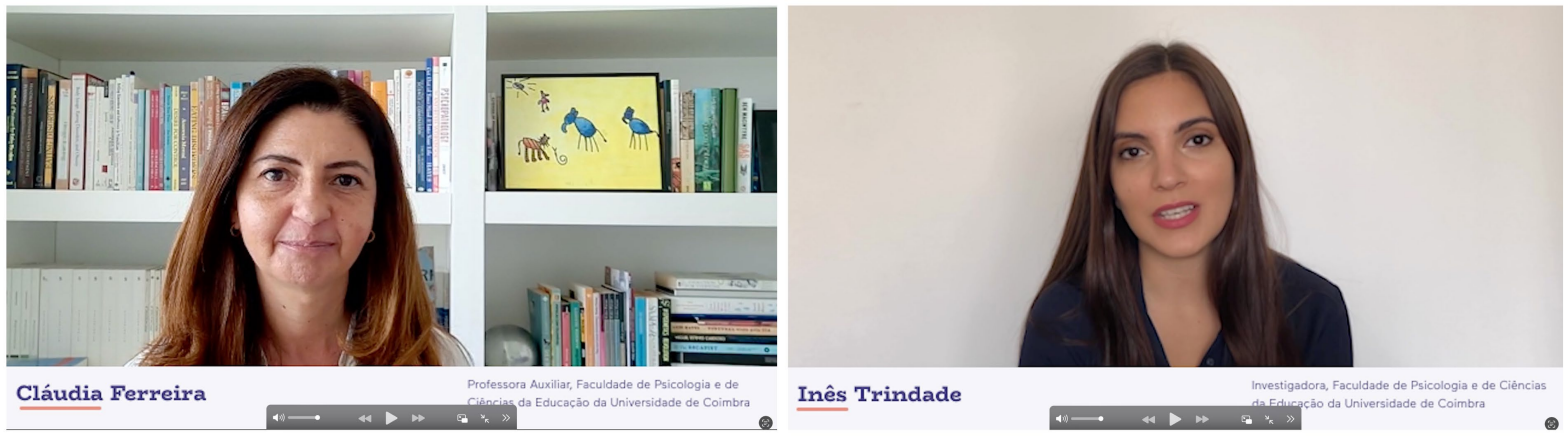
Real-life videos with the research team.

The eLIFEwithIBD intervention was delivered through 9 sessions that were available in the platform throughout at least a 9-week period. Each session comprises real-image videos, texts with illustrative images, exercises in editable text format, and audio files with experiential exercises and practices targeting the topic covered in the session (Figures 3, 4).

**Figure 5:**
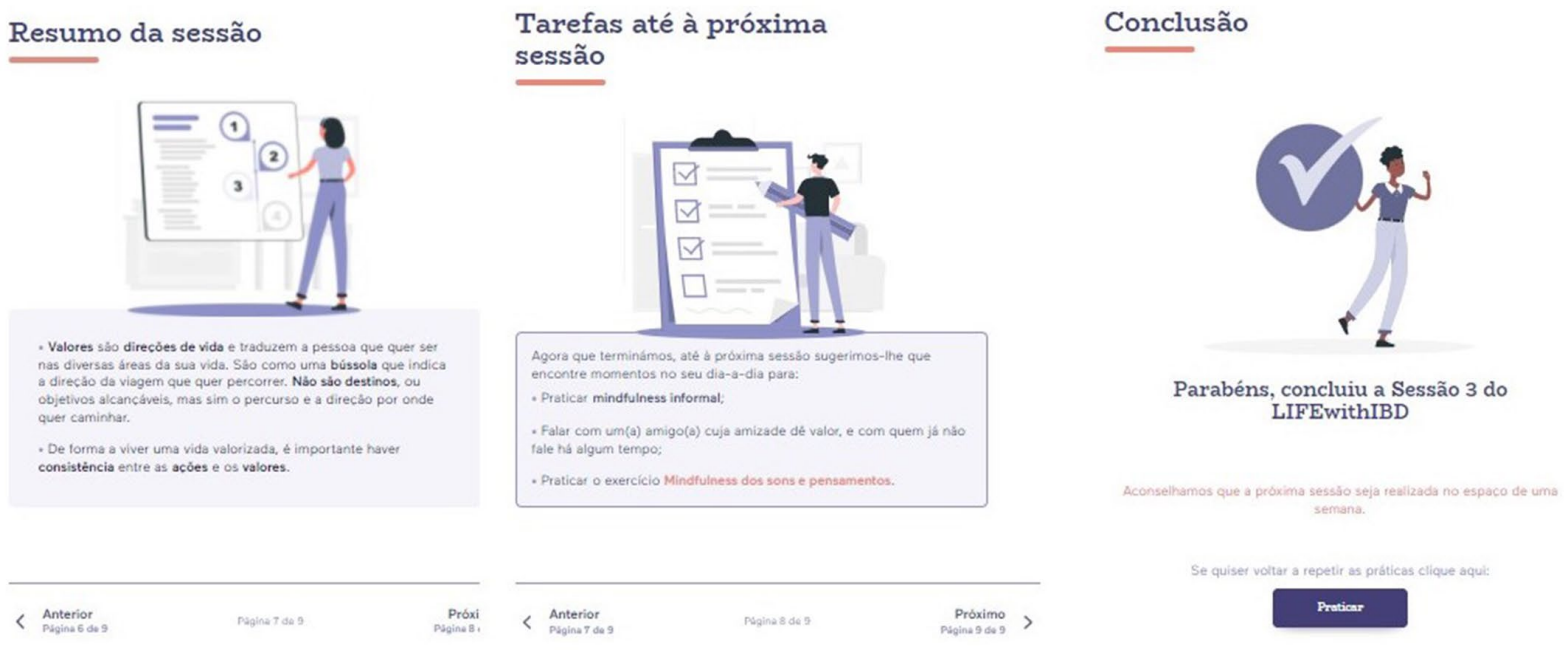
Example of the final pages of the sessions.

The first session of the eLIFEwithIBD intervention introduces the intervention’s purposes and structure, provides a rationale for the commonly identified areas of suffering, promotes creative hopelessness, and proposes mindfulness as a distinctive/alternative way of dealing with difficulties. Though addressing different contents, the subsequent sessions have a similar configuration: each session starts with a video in which a team member (researcher/clinician) welcomes the participant to the session, reviews the between-session assignments, and provides a summary regarding the topic that was covered in the previous session. Subsequently, the content of the current session is introduced, and experiential exercises are based on real-life situations of people with IBD are proposed. ACT metaphors and mindfulness or compassion meditation practices are also provided. At the end of each session (Figure 5), a summary of the session is presented, and assignments are prescribed for participants to do between sessions (e.g., mindfulness exercises and compassion practices available through audio files in the platform). These between-session assignments are aligned with the topics explored in each respective session.

**Figure 6:**
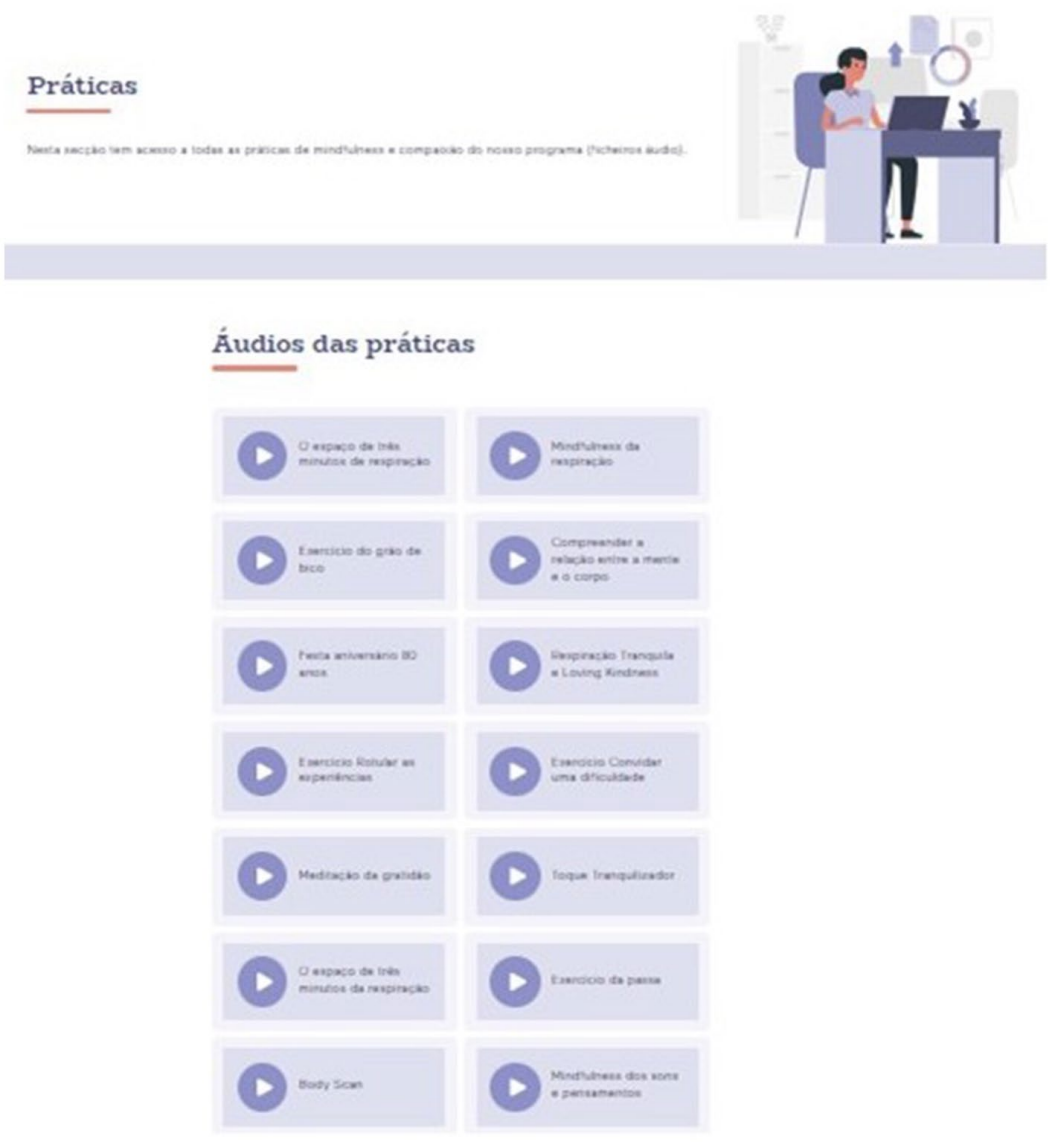
Practice section.

The audio files for the between-section meditation practice of that week would become available in the Practice section of the platform (Figure 6) after participants completed the respective session.

The final session (session number 9) includes a booster for committed action promotion (first covered in session 4), focusing on overcoming committed action barriers, and offers a wrap-up of the program. An overview of the entire intervention is presented in Table 1.

**Table 1:** eLifeWithIBD sessions, contents, practices, and exercises.

Session	Theme	Contents	Practices and exercises
0	Welcoming Creative hopelessness	Welcoming video Difficulty areas Introducing mindfulness Session summary	Difficulty areas exercise and feedback Three-minutes breathing space practice *Between sessions*: Informal mindfulness practices (e.g., mindful eating, mindful walking)
1	IBD psychoeducation	Introduction video Quiz comprising questions about IBD (exploring myths and common fears and worries) Session summary	Mindful Breathing *Between sessions*: Mindful Breathing practice; Informal mindfulness practices (e.g., mindful eating, mindful walking)
2	The evolutionary nature of the human mind and the functions of emotions	Introduction video What are the functions of human emotions (e.g., anxiety) Mind–body relationship video Emotion regulation through the body Session summary	Thought suppression (chickpea exercise) Bodyscan practice and feedback *Between sessions*: Bodyscan practice; Informal mindfulness practices (e.g., mindful eating, mindful walking)
3	Values clarification	Introduction video What are values? Exercise feedback Session summary	Passengers on the bus metaphor The 80-year-old birthday party Life areas exercise and feedback *Between sessions*: Talking to a good friend who has not spoken for some time; Informal mindfulness practices (e.g., mindful eating, mindful walking); Mindfulness of sounds and thoughts practice
4	Values and committed action barriers Self-care	Introduction video Defining aims and actions aligned with values Identifying barriers to committed actions Clarifying values Self-care: Dealing with fatigue and tiredness Physical tiredness The spoons theory Psychological tiredness Session summary	Aims and actions aligned with values exercise Barriers to committed actions exercise Values clarification exercise and feedback *Between sessions*: Informal mindfulness practices (e.g., mindful eating, mindful walking); Three-minutes breathing space practice three times a day
5	Compassion	Introduction video Shame and self-criticism Introduction to compassion Session summary	Loving-kindness practice and feedback *Between sessions*: Soothing breathing rhythm; Loving-kindness; Informal mindfulness practices (e.g., mindful eating, mindful walking)
6	Acceptance and cognitive defusion	Introduction video The power of thinking Labeling experiences Acceptance: What is it, and why is it important to promote it? Session summary	Guided imagery – Orange exercise Labeling experiences exercise and feedback Inviting a difficulty mindfulness practice and feedback *Between sessions*: Mindfulness of sounds and thoughts practice; Bodyscan practice; Doing daily activities in a different way and being in touch with unpleasant feelings; Informal mindfulness practices (e.g., mindful eating, mindful walking)
7	Gratitude	Introduction video Gratitude and mental health Promoting compassion and gratitude toward the body Session summary	Gratitude practice and feedback Soothing touch and feedback *Between sessions*: The ten fingers gratitude exercise daily practice; Compassionate body scan practice; Informal mindfulness practices (e.g., mindful eating, mindful walking)
8	eLifeWithIBD wrap-up	Introduction video eLifeWithIBD wrap-up Promoting committed actions	Obstacles in the river: Before vs. now exercise Three-minutes breathing space practice

### 2.4 Primary and secondary outcomes in the RCT

Participants completed self-report measures at three different times: baseline (T0), post-treatment (T1), and 4-month follow-up (T2) (Table 2).

**Table 2:** Schedule of enrollment, intervention, and assessments.

Study period					
	Enrollment	Allocation	Post-allocation		
Timepoint	-t2	-t1	t0	t1	t2
**Enrollment**					
*Informed consent*	x				
*Eligibility screen*	x				
*Blinded randomization*		x			
*Allocation*		x			
**Intervention**					
*eLIFEwithIBD*			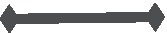		
*Waiting list*					
**Primary outcome**					
*DASS-21*			x	x	x
**Key mediators**					
*CompACT*			x	x	x
*SELFCS-SF*			x	x	x
**Secondary outcomes**					
*WSAS*					
*EUROHIS-QOL-8*			x	x	x
*IBDQ-UK*			x	x	x
*CISS*			x	x	x
*IBD Symptom Perception*			x	x	x
*Usability, acceptability and feasibility assessment*^a^				x	

a Only for the experimental condition.

DASS-21, Depression Anxiety Stress Scales; CompACT, Comprehensive assessment of Acceptance and Commitment Therapy processes; SELFCS-SF, Self-Compassion Scale Short Form; WSAS, Work and Social Adjustment Scale; EUROHIS-QOL-8, EUROHIS-QOL 8-item index; IBDQ-UK, Inflammatory Bowel Disease Questionnaire – UK version; CISS, Chronic Illness-related Shame Scale; IBD Symptom Perception, measured by the IBD symptoms scale.

#### 2.4.1 Primary outcome

##### 2.4.1.1 Psychological distress

The *Depression Anxiety Stress Scales* (DASS-21) (Lovibond and Lovibond, 1995; Bowen et al., 2009) is a 21-item widely used self-report instrument that assesses depressive (e.g., “I felt that life was meaningless”), anxiety (e.g., “I was aware of dryness of my mouth”), and stress (e.g., “I found it difficult to relax”) symptoms. Each item is rated on a 4-point scale. Higher scores denote higher psychological distress. The DASS-21 showed good reliability for the three subscales in its original (α_depression_ = 0.88; α_anxiety_ = 0.82; α_stress_ = 0.90) (Bowen et al., 2009) and Portuguese versions (α_depression_ = 0.85; α_anxiety_ = 0.74; α_stress_ = 0.81) (Lovibond and Lovibond, 1995).

#### 2.4.2 Mediators of change

##### 2.4.2.1 Psychological flexibility

The *Comprehensive assessment of Acceptance and Commitment Therapy processes* (CompACT) (Pais-Ribeiro et al., 2004; Trindade et al., 2021a,b,c) is a general measure of psychological flexibility as conceptualized by ACT. It comprises three dimensions of psychological flexibility: (i) openness to experience (e.g., “One of my big goals is to be free from painful emotions” - reverse item), behavioral awareness (e.g., “I rush through meaningful activities without being really attentive to them” - reverse item), and valued action (e.g., “I can identify the things that really matter to me in life and pursue them”). Items are rated on a 7-point scale ranging from *never true* (0) to *always true* (6). Higher scores indicate greater psychological flexibility. The CompACT showed good reliability in its original (Cronbach’s alphas ranging between 0.87 and 0.91) (Pais-Ribeiro et al., 2004). The Portuguese version of the CompACT includes 18 items and has showed Cronbach’s alphas varying between 0.77 and 0.88 (Trindade et al., 2021a,b,c).

##### 2.4.2.2 Self-compassion

The *Self-Compassion Scale Short Form* (SCS-SF) (Raes et al., 2011; Castilho et al., 2015) is a brief 12-items alternative to the long-form version of the Self-Compassion Scale (SCS) (Neff, 2003) for the assessment of self-compassion. Items are answered on a 5-point Likert scale, ranging from *almost never* (1) to *almost always* (5). The SCS-SF includes compassionate self-responding items which assess self-kindness (e.g., “I try to be understanding and patient toward those aspects of my personality I do not like”), mindfulness (e.g., “When something upsets me, I try to keep my emotions in balance”), and common humanity (e.g., “I try to see my failings as part of the human condition”). The SCS-SF also addresses uncompassionate self-responding, encompassing self-judgment (e.g., “I’m disapproving and judgmental about my own flaws and inadequacies”), isolation (e.g., “When I fail at something that’s important to me, I tend to feel alone in my failure”), and overidentification (e.g., “When I’m feeling down, I tend to obsess and fixate on everything that’s wrong”) items. Higher scores correspond to higher levels of self-compassion. The SCS-SF revealed good reliability (α ≥ 0.86) and a near-perfect correlation with the SCS long form (*r* ≥ 0.97 all samples) (Raes et al., 2011). The Portuguese version of the SCS also showed good internal consistency (α = 0.89) (Castilho et al., 2015).

#### 2.4.3 Secondary outcomes

##### 2.4.3.1 Functional impairment

The *Work and Social Adjustment Scale* (WSAS) (Mundt et al., 2002) is a brief, 5-item measure of perceived functional impairment in daily activities (e.g., work, family, interpersonal relations, social and private leisure activities, and home management). Items are answered on a 9-point scale, ranging from *not at all (0)* to *very severely (8)*. Higher scores reflect higher functional impairment. The WSAS presented good internal consistencies across several different health conditions (ranging between 0.70 and 0.94) (Pedersen et al., 2017). The WSAS’s psychometric properties, validity, and sensitivity to change have been established in several studies (Mundt et al., 2002). In Portuguese studies, the WSAS revealed good reliability (ranging between 0.87 to 0.93) (Carvalho et al., 2020;Trindade et al., 2021a,b,c).

##### 2.4.3.2 General quality of life

The *EUROHIS-QOL 8-item index* (Power, 2003; Pereira et al., 2013) is a quality-of-life self-report measure comprising 8 items (general health, energy, daily living activity, overall quality of life, finances, social relationships, self-esteem, and home) obtained from the WHOQOL-Bref (The WHOQOL Group, 1994). Each item is answered using a 5-point Likert scale (matching the response scales used in the WHOQOL-bref). Higher scores indicate higher levels of quality of life. The EUROHIS-QOL 8-item index study, conducted in 10 different countries, showed an overall Cronbach’s alpha of 0.83 (Schmidt et al., 2005). In the Portuguese validation study, the scale presented a Cronbach alpha of 0.83 (Pereira et al., 2013).

##### 2.4.3.3 Health-related quality of life

The *Inflammatory Bowel Disease Questionnaire – UK version* (IBDQ-UK) (Cheung et al., 2000) is the Anglicized 30-item version of the IBDQ (Guyatt et al., 1989; Irvine et al., 1994), an IBD-specific quality of life measure. The IBDQ-UK’s authors have modified the wording of some questions and simplified their response options. Items are rated on a 4-point scale ranging from “no, no at all/none” (0) to “on 8 – 14 days (i.e., more than every other day)/Yes, all of the time” (4). Respondents are asked about their experience with IBD and how it has impacted their lives during the preceding two weeks. The IBDQ-UK showed a very good reliability (Cronbach’s alpha of 0.94) in its original study (Cheung et al., 2000). A Portuguese version of this scale was created by the present research team for the purpose of the face-to-face LIFEwithIBD trial.

##### 2.4.3.4 Chronic illness-related shame

The *Chronic Illness-related Shame Scale* (CISS) (Trindade et al., 2017a,b) is a 7-item self-report instrument that specifically targets shame related to the experience of having a chronic illness and/or its symptoms (e.g., “I feel isolated/alone due to my illness,” “I feel inferior and disregard myself because of my illness,” “I feel inadequate because of my illness and symptoms”). Items are answered using a 5-point Likert scale ranging from *never true* (0) to *always true* (4). Higher scores suggest higher levels of chronic illness-related shame. In the original study, conducted in Portugal, the CISS showed a very good reliability (α = 0.91) (Trindade et al., 2017a,b).

##### 2.4.3.5 IBD symptom perception

The *IBD symptoms scale* (Trindade et al., 2017a,b) is a 16-item self-report scale designed to assess the perceived frequency of IBD symptoms during the previous month (e.g., fatigue, abdominal pain, bloating, flatulence, diarrhea, nausea or vomiting, fever, urgency). Items are rated on a 7-point scale, ranging from *never* (0) to a*lways* (6). Higher scores reveal higher levels of IBD symptom perception.

#### 2.4.4 Usability, acceptability, and feasibility assessment

Participants in the experimental condition evaluated the quality of the eLIFEwithIBD intervention through several questions regarding the platform (e.g., audio files), and their perceived change on several different aspects (e.g., IBD symptoms, ability to self-regulate emotions, quality of life, perception of change by close ones). The following validated self-report measures were used to evaluate the usability, acceptability, and feasibility of eLIFEwithIBD:

##### 2.4.4.1 System usability scale

The System Usability Scale (Brooke, 1996; Martins et al., 2015) is a 10-item self-report scale aimed to evaluate product and user interface usability (e.g., the confidence and ease of use of a platform). Items are rated on a 5-point scale ranging from *completely disagree* (1) to *completely agree* (5). Higher scores reveal more usability of a given digital product.

##### 2.4.4.2 Acceptability (Aim) and feasibility (FIM) of intervention measures

The Acceptability and Feasibility Measures (Weiner et al., 2017) are 4-item self-report instruments that assess the extent to which an intervention is acceptable (e.g., “the intervention meets my approval”) and feasible (e.g., “the intervention seems easy to use”). Items are answered using a 5-point Likert scale ranging from *completely disagree* (1) to *completely agree* (5). Higher scores reveal greater acceptability and feasibility. Portuguese translations of the AIM and FIM were developed by the current research team for the purpose of this study, using forward and back translation methods.

### 2.5 Statistical analysis

#### 2.5.1 Sample size

An *a priori* power analysis for this study was performed. In the absence of meta-analytic evidence, we have used data from Wynne and colleagues (Ungaro et al., 2017) where moderate-to-large reductions in psychological distress were observed after baseline adjustment as a result of an ACT intervention in IBD. Given that there is little evidence to suggest the inferiority of internet-delivered interventions, we powered for an expected reduction in psychological distress of SMD = 0.75. Assuming an alpha of 0.05 and 80% power, we estimate that a total sample size of n = 58 would be required to power the primary analysis.

### 2.6 Planned analyses

#### 2.6.1 Data analysis

All analyses are planned as intent-to-treat (ITT) and will be examined using Jamovi statistical software (Jamovi, 2020), and R (R Core Team, 2021).

#### 2.6.2 Primary outcome analysis

The primary outcome will be the psychological distress (DASS-21–depression, anxiety, and stress) measured at 9 weeks (immediately post-intervention) between the two intervention groups and adjusting for baseline scores using ANCOVA. We will examine the ANCOVA’s assumptions through standard tests and residual plots and perform transformations. The magnitude of the differences between-treatment groups will be expressed as the standardized mean difference.

#### 2.6.3 Secondary analyses

Between-groups differences at 9 weeks will be examined for the following continuous outcomes: IBD symptom perception (IBDSS); psychological processes (CompACT, SCS-SF); chronic illness-related shame (CISS); work and social adjustment (WSAS); and quality of life (EUROHIS-QOL-8, IBDQ-UK). All analyses are to be conducted using ANCOVA with baseline values as a covariate.

#### 2.6.4 Longitudinal outcomes

We will use Mixed Models of Repeated Measures (MMRM) to examine for Group Time interaction effects on each of the continuous outcomes from baseline, 9 weeks and 4 months; the DASS-21, the IBD symptom perception scale; SCS, CompACT, CISS, WSAS, EUROHIS-QOL-8, and IBDQ-UK.

#### 2.6.5 Exploratory outcomes

Reliable change indices between baseline and follow-up points will be calculated for all outcomes and correlated with demographics to examine for subgroup efficacy predictors. The cut-off for clinically significant change is generally accepted as ±1.96 (R Core Team, 2021). For exploratory analyses focused upon proportionate data, such as comparisons of mood dysfunction outcomes, McNemars’ test can be used to examine within-group differences across time points.

#### 2.6.6 Attrition and protocol non-adherence

In accordance with ITT principles, all participants recruited into the study, regardless of treatment group, are to have as much data as possible recorded as fully as possible in accordance with this protocol. All cases of missing data, including non-retention of participants or withdrawal of consent, are to be recorded by the research staff. Where data is missing, the frequency and reasons for missing data are to be recorded. Missing data is to be statistically examined for patterns of systematic missingness.

In situations where data is missing, the data collected up to that point will be used, provided the participant has not withdrawn their consent or requested that the information be removed.

## 3 Discussion

This trial will be the first to examine the acceptability, usability, and preliminary efficacy of an online ACT, mindfulness, and compassion-based intervention on the improvement of psychological distress and other psychosocial indicators in IBD. This study is in line with the current directions from the Portuguese e-health Strategy (SPMS, 2015), the European e-Health Action Plan 2012–2020 (European Commission, 2012), and (World Health Organization, 2021) in what regards the promotion of the use of web-based technology in clinical practice and to improve patient-centered care and the efficiency of health systems.

The inclusion of an inactive control will be a limitation to this RCT, particularly given that participants were aware of their non-assignment to the experimental group, which may influence responses to self-report questionnaires. Similarly to previously conducted online interventions (Wahbeh et al., 2014), this study may include low adherence and high attrition rates. In fact, participants’ engagement tends to be lower when enrollment is entirely online, interventions are self-guided, and long (> 8 weeks) (Linardon and Fuller-Tyszkiewicz, 2020).

## 4 Conclusion

The current trial aims to contribute to a shift in IBD healthcare from a sole focus on medical or physical indicators to a more comprehensive approach which also comprises the promotion of psychological health while taking advantage of the potential of e-health tools. Our study’s strengths include the use of a randomized controlled design and well-accepted validated self-report questionnaires. This feasibility trial could be the starting point for future studies (e.g., with larger samples, assessing cost-effectiveness) to explore the effects of online interventions as a complement to the regular healthcare of IBD patients, to improve quality of life and mental health as well as to reduce long-term medical costs associated with IBD.

## Ethics statement

The studies involving humans were approved by the Faculty of Psychology, University of Coimbra, Portugal. The studies were conducted in accordance with the local legislation and institutional requirements. The participants provided their written informed consent to participate in this study. The individual(s) provided their written informed consent for the publication of any identifiable images or data presented in this article.

## Author contributions

CF: Conceptualization, Funding acquisition, Investigation, Methodology, Project administration, Resources, Supervision, Validation, Writing – original draft. JP: Data curation, Formal analysis, Investigation, Methodology, Writing – original draft. IM-P: Data curation, Formal analysis, Investigation, Methodology, Writing – original draft. DS: Data curation, Formal analysis, Investigation, Writing – original draft. AG: Methodology, Validation, Writing – original draft. NF: Conceptualization, Investigation, Writing – review & editing. SC: Investigation, Resources, Writing – review & editing. PL-S: Writing – review & editing. BR: Writing – review & editing. SO: Writing – review & editing. FP: Conceptualization, Investigation, Writing – review & editing. IT: Conceptualization, Funding acquisition, Investigation, Methodology, Project administration, Resources, Supervision, Validation, Writing – review & editing.
